# Automated conduction velocity estimation based on isochronal activation of heart chambers

**DOI:** 10.1007/s10840-022-01339-1

**Published:** 2022-09-30

**Authors:** Michela Santurri, Jennifer Bonga, Maurizio Schmid, Filippo Maria Cauti, Francesco Solimene, Marco Polselli, Mauro Bura, Francesco Piccolo, Maurizio Malacrida, Gemma Pelargonio, Francesco Raffaele Spera, Stefano Bianchi, Pietro Rossi

**Affiliations:** 1 BioLab3, Biomedical Engineering Laboratory, Roma Tre University, Rome, Italy; 2 Arrhythmology Unit, Hospital Fatebenefratelli Isola Tiberina-Gemelli Isola, Rome, Italy; 3 Electrophysiology Unit, Clinica Montevergine, Mercogliano, Avellino Italy; 4 Boston Scientific, Milan, Italy; 5 Cardiovascular Sciences Department, Fondazione Policlinico Universitario Agostino Gemelli IRCCS, Rome, Italy

**Keywords:** Conduction velocity, Isochronal activation time, Geodesic distance, Arrhythmias, Slow conduction, Cardiac mapping

## Abstract

**Background:**

Spatial differences in conduction velocity (CV) are critical for cardiac arrhythmias induction. We propose a method for an automated CV calculation to identify areas of slower conduction during cardiac arrhythmias and sinus rhythm.

**Methods:**

Color-coded representations of the isochronal activation map using data coming from the RHYTHMIA™ Mapping System were reproduced by applying a temporal isochronal window at 20 ms. Geodesic distances of the 3D mesh were calculated using an algorithm selecting the minimum distance pathway (MDP). The CV estimation was performed considering points on the boundary of two spatially and temporally adjacent isochrones. For each of the boundary points of a given isochrone, the nearest boundary point of the consecutive isochrone was chosen, the MDP was evaluated, and a map of CV was created. The proposed method has been applied to a population of 29 patients.

**Results:**

In all cases of perimitral atrial flutter (16 pts out of 29 (55%)), areas with significantly low CV (< 30 cm/s) were found. Half of the cases present regions with low CV located in the anterior wall. No case with low CV at the so-called LA isthmus was observed. Right atrial maps during common atrial flutters showed low CV areas mainly located in the inferior inter-atrial septum. No areas of low CV were observed in subjects without a history of atrial arrhythmia while pts affected by paroxysmal AF showed areas with a limited extension of low CV.

**Conclusions:**

The proposed software for automated CV estimation allows the identification of low CV areas, potentially helping electrophysiologists to plan the ablation strategy.

## Introduction

Spatial heterogeneity in CV across the myocardium is critical for the induction and maintenance of cardiac arrhythmias [1–4].

Several methods for spatially estimating tissue CV [1, 4–8] have been developed in order to improve our understanding of arrhythmogenic substrate and to identify an effective target for ablative therapy [9, 10].

Isochronal maps have long been regarded as the gold standard for evaluating tissue activation uniformity in animal and human models [10–12], during sinus rhythm (SR) or arrhythmias [13].

However, despite the actual availability of many methods for CV estimation, there is still an active debate in this field, and no consensus has been reached on the optimal strategy to obtain relevant clinical information from CV estimation.

The present study aims to introduce an automated CV calculation method, based on isochronal activation timings, which helps to better identify areas of slower conduction displayed on a 3D electro-anatomic map (EAM) of the heart chambers during atrial and ventricular arrhythmias.

## Methods

### Electroanatomic mapping data

High-density EAMs of different cardiac chambers were obtained using the RHYTHMIA™ Mapping System and a 64-pole mapping catheter, the INTELLAMAP ORION (Boston Scientific Corp.,) which incorporates 64 printed minielectrodes (surface area 0.4 mm^2^, spacing 2.5 mm). Two reference electrograms (EGMs) are chosen from the multipolar diagnostic catheter (Polaris X, Boston Scientific Corp) positioned in the coronary sinus (CS). Cardiac beats are automatically selected for inclusion in the map based on a predefined set of acceptance criteria as described by Bollmann et al. [14]. The system sets the temporal window of interest’s width at the cycle length value and centers it on the main reference EGM. Local activation time is annotated by taking into account the maximum voltage amplitude of each bipolar signal. For fragmented EGMs with multiple signal components, the system takes into account the coherence of the annotation with that of the points in the surrounding area. The chamber surface geometry is generated using the location of the outermost electrodes, and it is constantly updated during mapping. Selection of the surface EGMs is based on the projection distance (which can be varied between 1 and 5 mm): only EGMs recorded within the projection distance from the surface geometry are displayed.

The system lets the operator see the specific location of the ORION mapping catheter in the EAM, making it possible to build an anatomical map of the cardiac chamber in real time. The activation time of the electrograms recorded in different locations on the cardiac wall is extracted by recording the electrical activity and estimating the timing of each acquired point by annotation compared to the reference signal. The EAM is then created by integrating the anatomical map with the spatially referenced electrical activity and each annotated point.

The electrical section of the map is composed of projected surface electrodes, which are defined as the locations of the electrodes of the basket catheter when they are less than 2 mm away from the chamber walls. Electrical activity is then assigned to each vertex of the anatomical map based on the activation of the surface electrode closest to it. The EAM thus obtained will be used for the following phase of the CV estimation algorithm.

### Isochronal map area definition

From the EAM, the previously described dataset is subdivided into different regions, called isochronal map areas, based on activation timings falling within a specified temporal timeframe (temporal isochronal window, TIW).

A color-coded representation of the areas associated with the different isochrones makes it possible to qualitatively capture and visualize the propagation of activation.

To quantify the amount of propagation and estimate the conduction velocity, it is necessary to introduce the distance traversed by the propagation wave across successive regions. If the TIW size is constant for every region, the size of a region can be linked to conduction velocity, which can be calculated as the ratio of the traveled distance to the time needed to traverse it: in faster conduction regions, propagation covers a greater distance in the same amount of time, whereas in slower propagation ones it traverses a smaller one [15].

### Geodesic distances

Each isochronal map area is generally characterized by two separated boundaries, which connect to the temporally preceding and following areas, respectively. Given an isochronal map area, starting vertices are identified as those at the boundaries with the preceding area, while arrival candidate vertices are those at the boundaries with the following one. For each starting vertex, we determine a correspondence with one arrival candidate as the vertex at the margin of the following map areas yielding the closest Euclidean distance from the starting vertex. If the identified arrival vertex does not pertain to a temporally consecutive isochronal map area, it means that an isochronal jump occurs, configuring a condition of the local block. If the TIW is set to 20 ms, an isochronal jump is present when the temporal distance between timing annotations of starting and arrival vertices is higher than 40 ms. This particular situation occurs when the vertices on the boundary of two isochronal map areas are adjacent in space but not consecutive in time.

**Fig. 1:**
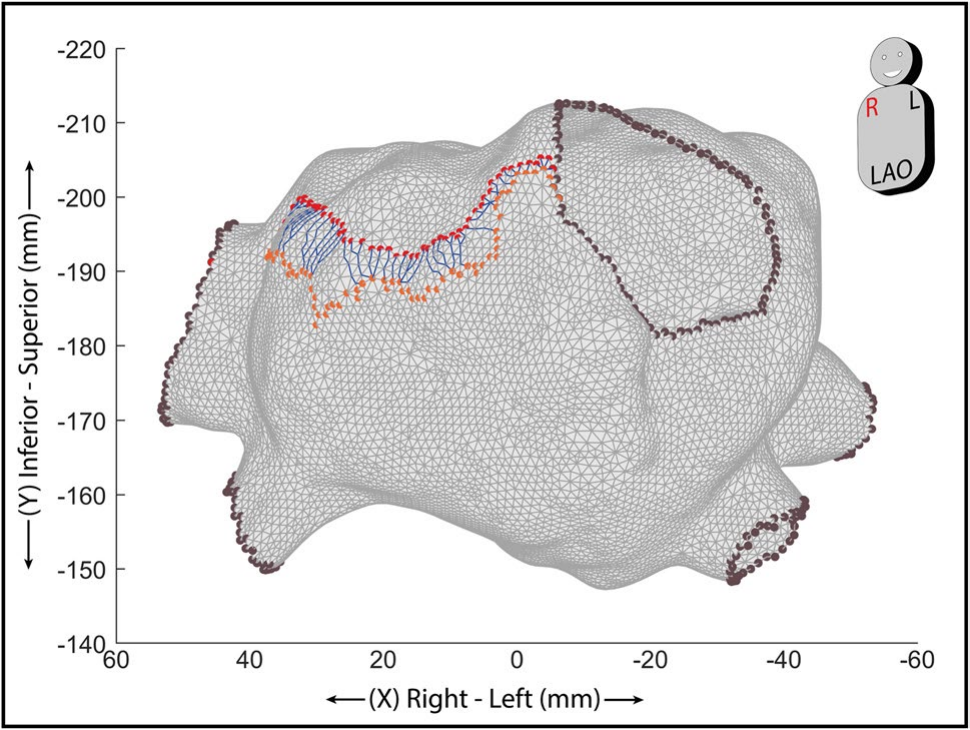
Technique for the definition of minimum distance pathways. The overall anatomical map is displayed in gray (vertices corresponding to anatomical cuts are displayed in brown). Starting vertices of one isochronal map area are displayed in red, and arrival vertices are displayed in orange. For each starting vertex, the minimum distance pathway obtained according to the procedure described in the text is highlighted with a blue line. Multiple pathways may merge toward one common arrival vertex. While no direct constraints are given on the perpendicularity of the path with respect to the boundary, the implementation of the minimum path distance described in the text makes the path proceed toward the arrival vertex, thus indirectly granting a tendency to normality with respect to the arrival vertex

In this study, we introduce a method based on Dijkstra’s [16] for the calculation of the travelled distance between each vertex pair when starting and arrival vertices pertain to temporally and spatially consecutive isochronal map areas. Beginning from the starting vertex (*p*_*i*_, *i* = 1), the successive one (*p*_*i* + 1_) is chosen among all the vertices directly connected to the starting one, as the one at the shortest Euclidean distance from the arrival vertex. All other candidate vertices are thus rejected, and will not be considered further among the candidates for the successive iterations. The process is then iterated by considering p_i+1_ as the current starting one, and by repeating the selection among remaining candidates using the same criterion. By iterating this process toward the arrival point, a minimum distance pathway is drawn for each pair, and its distance MDP is then determined as follows:

$$\mathrm{MDP}={\sum }_{i=1}^{N-1}\left|\left({s}_{{p}_{i+1}}-{s}_{{p}_{i}}\right)\right|$$

where *N* is the number of the vertices that compose each pathway, *s*_pi_ is the spatial location of the generic vertex included in the minimum distance pathway, *s*_p1_ and *s*_pN_ are the locations of the starting and arrival vertex, respectively; and consecutive vertices pertain to the same triangular mesh. The output of the process for MDP calculation for one isochronal map area is displayed in Fig. 1.

Once all the MDPs are identified, the CV estimation follows.

### CV estimation

Conduction velocity is calculated by the formula below, under the assumption that the value of conduction velocity does not vary between points composing the minimum distance pathway:

$$\mathrm{CV}=\frac{\mathrm{MDP}}{\mathrm{TIW}}$$

We decided to estimate CV using vertices on the boundary of two adjacent and temporally consecutive isochronal map areas; for each point of the isochronal map area boundary, the nearest boundary point of the successive isochronal map area is identified using the process described before, and the corresponding CV value is then assigned to all the points composing each pathway. To increase readability in identifying spatial differences of estimated CV, no interpolation of CV was performed for those inner points of each isochronal map area that were not assigned to any minimum distance pathway.

This process is then iterated among consecutive temporal isochronal map areas, thus building a map for CV estimation of the involved cardiac chamber.

### Map settings for clinical studies

Once the CV is calculated on all possible MDPs and isochronal map areas, to remove spurious outliers, only MDPs with CV values lower than 200 cm/s are maintained (values higher than this limit are rarely reported in both physiological and pathological cases). A color-coded representation is then used, with intervals spaced of 5 cm/s in the range from 0 to 30 cm/s, to visually identify possible deceleration zones. The upper limit of CV can be set manually by physicians through a slider. Additionally, graphical representations of lines of blocks are added (black points in the figures), defined as spatial zones on the surface of the involved cardiac chamber where a temporal gap, from the starting and arrival points of adjacent isochrones, was at least 40 ms when the TIW is fixed at 20 ms (2 * TIW). The cutoff value of 30 cm/s was chosen according to ranges described in previous papers [17, 18].

The map is automatically acquired by the mapping system, which can filter out inconsistent EGMs as indicated in the references [14, 19, 20]. The maps were examined by a mapping specialist to remove acquired surface EGMs with incorrect annotations that were inconsistent when compared with the surrounding ones. These can be rapidly identified as surface electrode locations displaying a color different from that of the isochronal area within which they fall.

### Sensitivity evaluation

To identify which TIW size best suited the clinical outcomes of each case, four different values of TIW were tested (10, 20, 30, and 40 ms). We calculated the estimated CV according to each TIW size and performed a one-way analysis of variance on the estimated velocities with TIW size as a factor, to check whether there is an effect coming from its choice.

### Study population

We applied the proposed methodology to 29 patients. A subgroup of 16 out of 29 pts (55%) with perimitral flutter; 4 pts (14%) affected by common right atrial flutter; 4 pts (14%) suffering from paroxysmal atrial fibrillation (AF); 1 pt affected by VT (3%) and 4 pts (14%) underwent to left accessory pathways ablation as the control group).

## Results

### Slow conduction areas during sinus rhythm

**Fig. 2:**
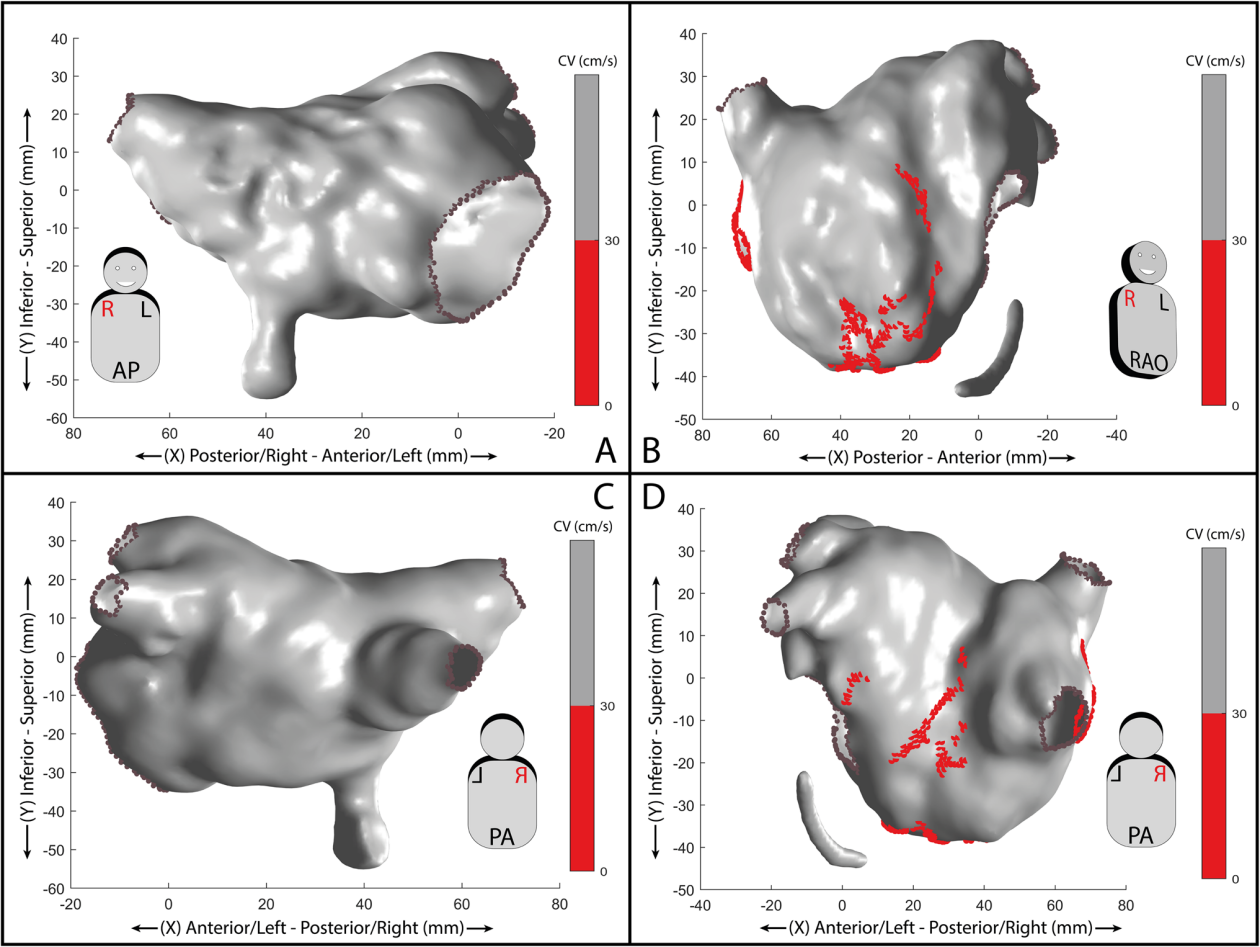
**A** Conduction velocity (CV) 3D map of a left atrium in a patient without AF history, anterior wall (antero-posterior, AP view). No slow conduction zones (< 30 cm/s) are present. **B** Conduction velocity (CV) 3D map of a left atrium in a patient with AF history, anterior wall (right anterior oblique, RAO view). Slow conduction zones with CV lower than 30 cm/s are displayed in red. **C** Conduction velocity (CV) 3D map of a left atrium in a patient without AF history, posterior wall (postero-anterior, PA view). No slow conduction zones are present. **D** Conduction velocity (CV) 3D map of a left atrium with AF history, posterior wall (PA view). Slow conduction zones with CV lower than 30 cm/s are displayed in red. In all panels, each axis’ values are expressed as millimeters

In the 4 pts who underwent a left accessory pathway and without a history of atrial arrhythmias, no areas of low CV were observed. A representative case is reported in Fig. 2 panels A and C.

Areas with slow CV (≤ 30 cm/s) were found in the LA of a subject with a history of atrial arrhythmias. Figure 2 panels B and D show the LA map of a patient affected by paroxysmal AF, revealing limited areas of slow CV located either at the anterior and posterior wall or in the septal region. The mean CV of the healthy LA was 96 ± 49 cm/s while 80 ± 46 cm/s for the diseased LA.

### Subgroup population with atrial flutters

In all cases of perimitral atrial flutter, LA areas with significantly low CV (< 30 cm/s) were found. The regions of the LA with slow CV were the anterior wall in 8 out of 16 pts (50%); the anterior and posterior wall in 7 out of 16 (44%) and only the posterior wall in 1 out of 16 pts (6%). In no case, low CV was observed at the so-called LA isthmus (the region between the right inferior pulmonary vein and the lateral segment of the mitral ring).

Areas of low conduction were found also in the right atrium in all pts during common atrial flutters. In these cases, the low CV areas were mainly located in the inferior interatrial septum between the segment of the tricuspidal annulus near the coronary sinus ostium and the inferior vena cava.

The details of three reentrant circuits (one typical atrial flutter, one peri-mitral atrial flutter, and one reentrant ventricular tachycardia originating from the left ventricle) were reported as significant examples of computed CV and its spatial distribution. In all reported cases, the entire tachycardia cycle length could be endocardial mapped. The anatomical location of slow CV in the analyzed atrial flutters are reported in Table 1.

**Table 1:** Low CV (< 30 cm/s) during typical and atypical flutter study cases

	Location 1 with CV < 30 cm/s	%	Location 2 with CV < 30 cm/s	%	Location 3 with CV < 30 cm/s	%
Atypical atrial flutter (*n* = 16)	AW	50 (8/16)	AW + PW	44 (7/16)	PW	6 (1/16)
Typical atrial flutter (*n* = 4)	IAS	75 (3/4)	IAS + IVC-TA isthmus	25 (1/4)	-	-

This table reports the most common locations of low CV (< 30 cm/s) during typical and atypical flutter study cases. *AW* anterior wall, *PW* posterior wall, *IAS* interatrial septum, *IVC-TA* inferior vena cava-tricuspidal annulus

### Representative case of typical atrial flutter circuit

The EAM of the right atrium was performed during a typical (peritricuspidal) atrial flutter (AFL).

**Fig. 3:**
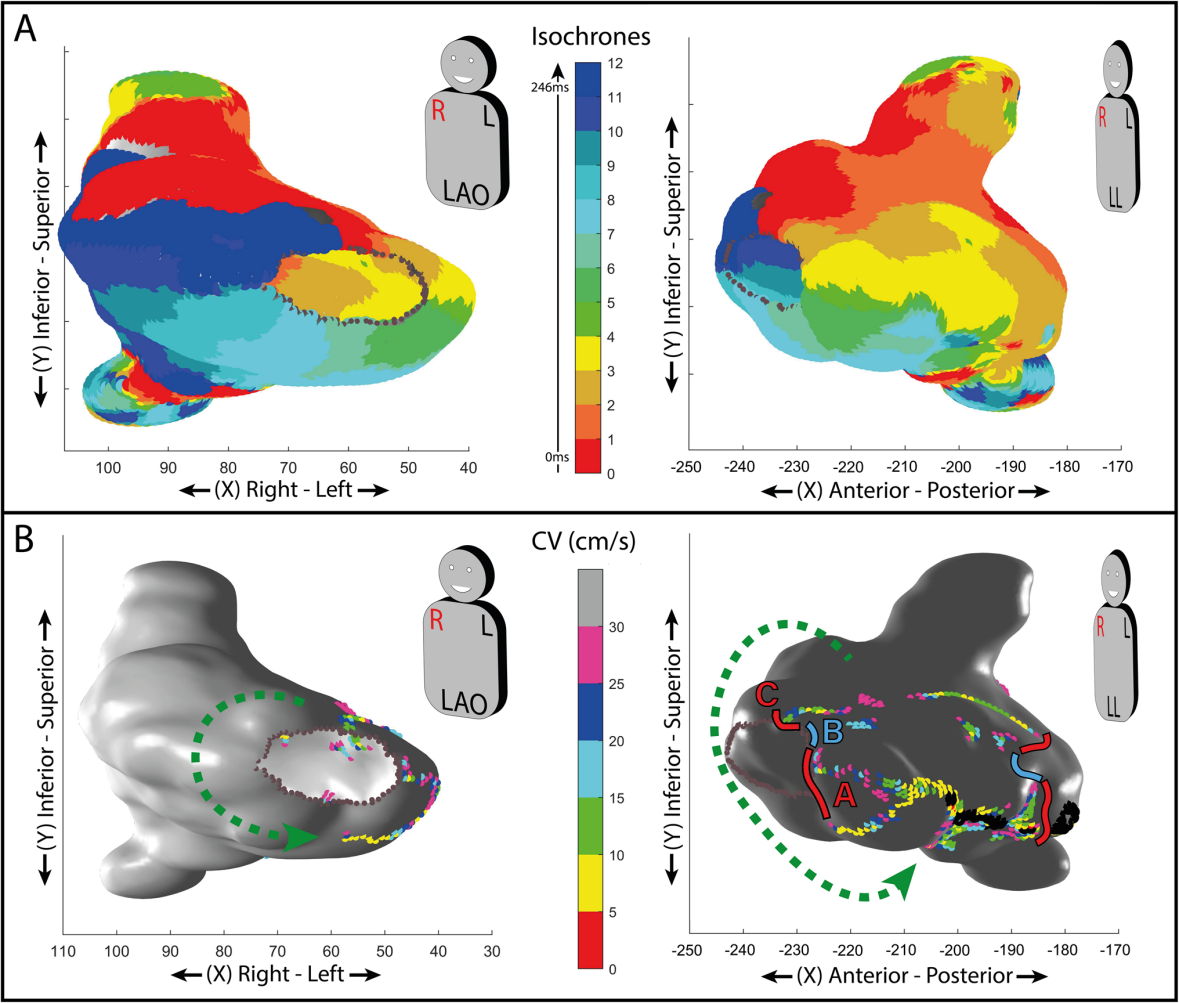
**A** Atrial isochronal 3D activation map of peritricuspidal (typical) atrial flutter (AFL), LAO and LL views. **B** Conduction velocity (CV) 3D map during perimitral AFL, LAO, and LL views. The alternating sequence of slow and faster conduction zones inside the isthmus is reported. Black dots highlight conduction block lines. Green, dotted arrows depict the flutter’s circuit—the “outer loop” portion. In both panels, each axis’ values are expressed as millimeters. A, B, and C letters highlight the three different zones: a slow lower part (**A**), a fast central part (**B**), and a slow upper part (**C**) of the interatrial septum

In this case, a total of 12 isochrones were defined. A map of the spatial distribution of CV for the left atrium was created as reported in Fig. 3 panel B. The same CV upper limit was equally set for all the examples reported, at 30 cm/s, with 6 intermediate steps (5 cm/s each) for the color-graded scale.

The activation map of the right atrium demonstrated a peri-tricuspid AFL with a CL of 246 ms, as can be seen in Fig. 3 panel A. The automated analysis of the spatial CV distribution identified a line of conduction block in the lower region of the inter-atrial septum (black points in Fig. 3 panel B), sparing the area directly beneath the tricuspid annulus. In the septal zone, the first area of deceleration (Fig. 3 panel B—“A” zone), the second area of acceleration (Fig. 3 panel B—“B” zone), and the third area of deceleration going from the lowest to the highest part of the interatrial septum (Fig. 3 panel B—“C” zone) were present. Creating a block line by ablating from the vena cava to the tricuspid annulus led to AFL interruption and non-inducibility.

### Representative case of left atrial flutter circuit

The EAM of the left atrium was performed during AFL. The cycle length of AFL was 265 ms.

**Fig. 4:**
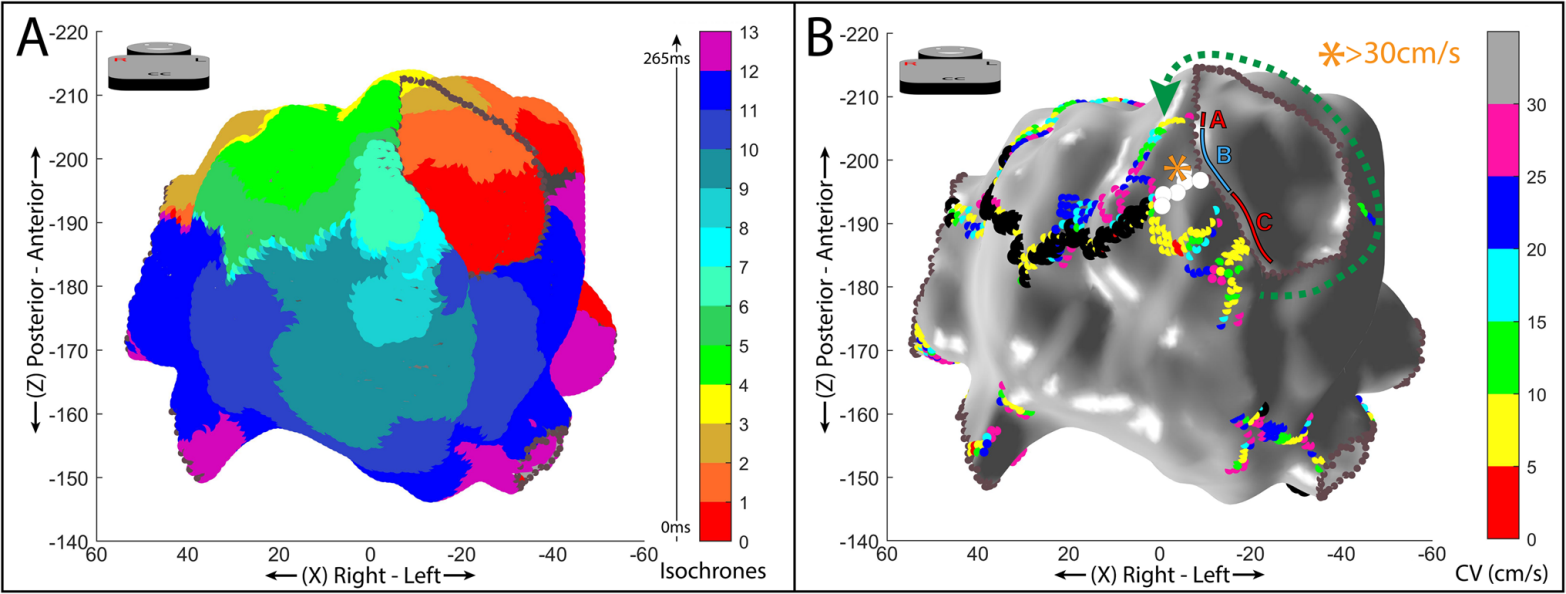
**A** Atrial isochronal 3D activation map of perimitral atrial flutter (AFL). **B** Conduction velocity (CV) 3D map during perimitral AFL. The alternating sequence of slow and faster conduction zones inside the isthmus is reported. The yellow asterisk is positioned in the area with higher CV (> 30 cm/s) included between two areas with CV < 30 cm/s. Black dots highlight conduction block lines. Green, dotted arrows depict the flutter’s circuit—the “outer loop” portion. Big white dots represent the ablation line which led to AFL interruption. In both panels, each axis’ values are expressed as millimeters. A, B, and C letters highlight the three different isthmus zones: a slow entrance (A), a fast central part (B), and a slow exit (C)

In this case, a total of 13 isochrones were defined. A map of the spatial distribution of CV for the left atrium was created as reported in Fig. 4 panel B. The same ventricular CV upper limit was set in the atrium, at 30 cm/s, with 6 intermediate steps (5 cm/s each).

The activation map of the left atrium demonstrated a peri-mitral AFL with a CL of 265 ms, as can be seen in Fig. 4 panel A. The automated analysis of the spatial CV distribution identified a septal line of conduction block (black points in Fig. 4 panel B) sparing the area proximal to the mitral annulus segment sustaining the AFL circuit. In this atrial zone, the first area of deceleration (Fig. 4 panel B – “A” zone with a mean CV of 11.69 ± 6.42 cm/s), the second area of acceleration (Fig. 4 panel B—“B” zone) with a mean CV of 55.10 ± 20.76 cm/s), and the third area of deceleration (Fig. 4 panel B—“C” zone with a mean CV of 18.03 ± 7.73 cm/s) were present. Extending the preexisting block line (black points) to the mitral annulus and extending lesions to the slow CV area with ablation, led to AFL interruption and non-inducibility.

### Representative case of ventricular tachycardia circuit

**Fig. 5:**
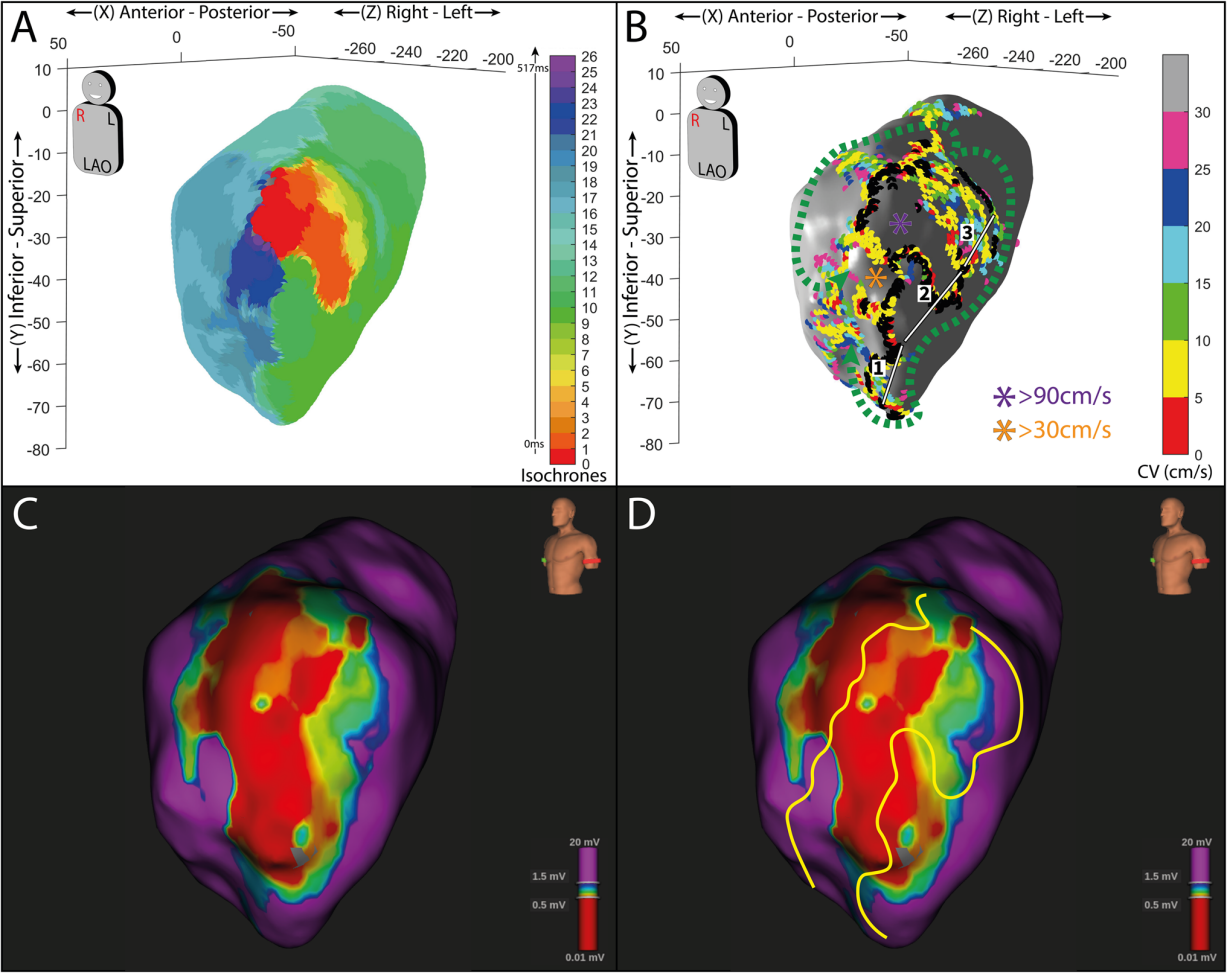
**A** Ventricular isochronal 3D activation map with 30° left anterior oblique (LAO) orientation. **B** Conduction velocity (CV) 3D map, with 30° left anterior oblique (LAO) orientation. White lines and numbers from 1 to 3 indicate the areas of the critical isthmus’: entrance (area 1), mid isthmus (area 2), and exit (area 3). An alternating sequence of slow and fast CV zones is found inside the critical isthmus channel of the VT circuit. Asterisks on gray areas identify, respectively, an area (orange asterisk) with a mean CV > 30 cm/s, and an area (purple asterisk) with a mean CV > 90 cm/s. Green, dotted arrows depict the tachycardia’s circuit—the “outer loop” portion. In both panels, each axis’ values are expressed as millimeters. **C** and **D** a voltage map of the same case, obtained with rhythmia system during sinus rhythm, with 30° left anterior oblique (LAO) orientation (panel C). In panel D, a superimposition of VT isthmus borders is shown on the voltage map. The color scale of the map in both panels represents voltages between 0.5 and 1.5 mV

The EAM of the ventricle was performed during ventricular tachycardia (VT). The cycle length (CL) of VT was 517 ms, and using a TIW of 20 ms, a total of 26 isochronal activation areas were defined (Fig. 5 panel A). A left ventricle map of CV spatial distribution was created as reported in Fig. 5 panel B. The upper limit of CV is set at 30 cm/s with 6 intermediate steps (5 cm/s each).

As it can be seen from Fig. 5 panel B, the spatial distribution of CV along the endocardial ventricle surface identifies the area of the critical isthmus of the circuit. The method automatically reports lines with wavefront conduction blocks (black points). The activation map allowed identifying and spatially segmenting entrance, central, and exit zones of the isthmus area (Fig. 5 panel B), while the CV map supplies information on the velocity of propagation wavefront, also highlighting the heterogeneity of CV in these three zones as assessed by the color-coded dots. The mean CVs were, respectively, (8.84 ± 6.03) cm/s at the entrance area and (9.35 ± 5.49) cm/s at the exit area. Inside the isthmus, an alternating sequence of acceleration/deceleration regions was observed as reported in Fig. 5 panel B where the CV value is (91.29 ± 35.95) cm/s. VT interruption was obtained by ablation at the isthmus area with the shortest transversal diameter.

### Sensitivity evaluation

We analyzed the impact of the CV calculation at different TIW sizes (10, 20, 30, and 40 ms) computing the data of the global heart chamber map, and no significant differences are found.

**Fig. 6:**
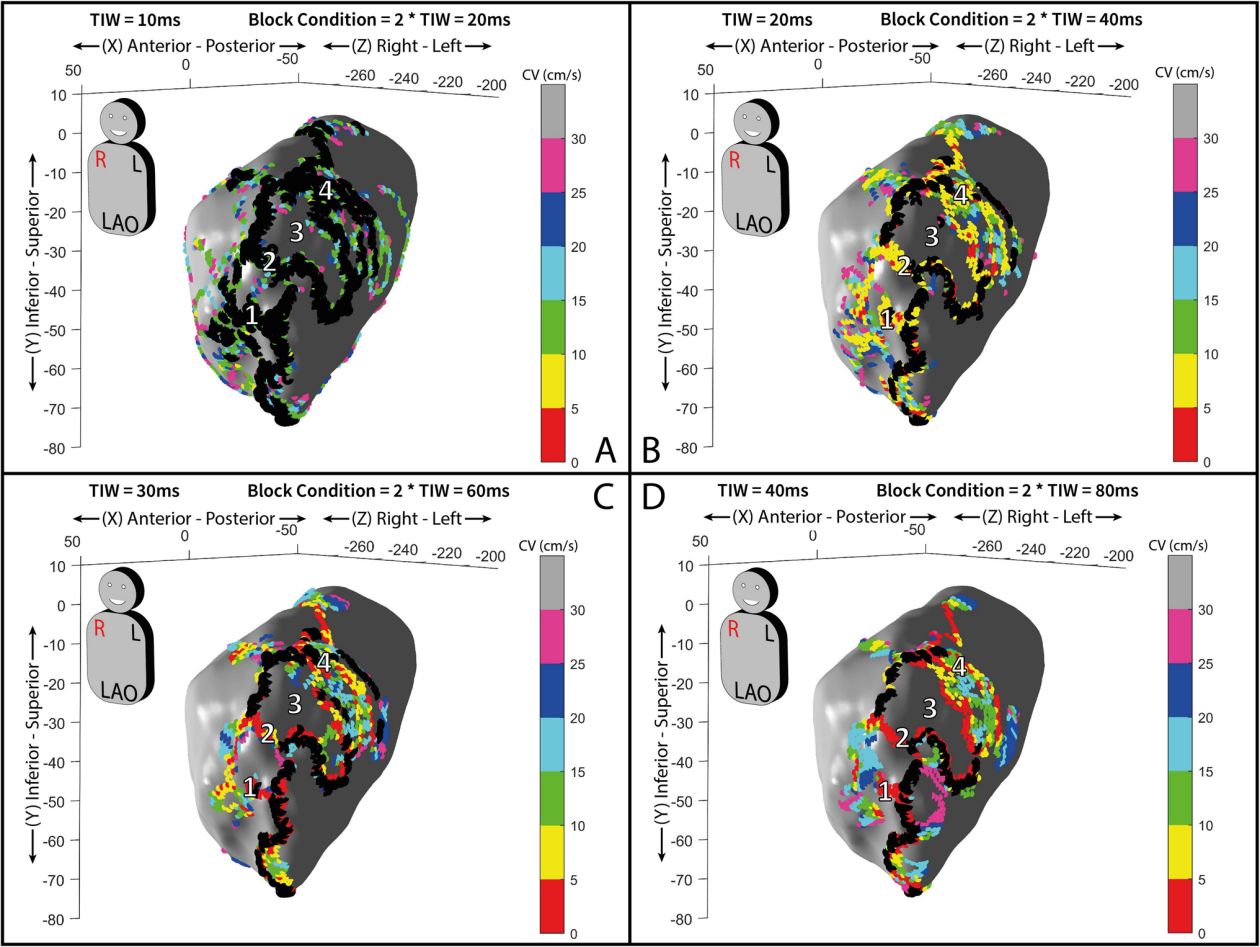
**A** to **D** 3D maps of conduction velocities (CV) set at different TIW sizes (A: TIW = 10 ms, B: TIW = 20 ms, C: TIW = 30 ms, D: TIW = 40 ms) with 30° left anterior oblique (LAO) orientation. Inhomogeneous CV inside the isthmus channel of the VT circuit is reported in the images. The numbers 1, 2, and 4 are located in areas with low CV while number 3 identifies area with faster CV (> 30 cm/s). Color-coded dots show areas with slow (< 30 cm/s) and different CVs. Black dots highlight conduction block lines. Each axis’ values are expressed as millimeters. White numbers, from 1 to 4, in each panel indicate the different isthmus CV areas and their consistency across the different maps’ TIW sizes chosen, as n. 1, 2, and 4 are always slow and *n*. 3 is always fast

**Fig. 7:**
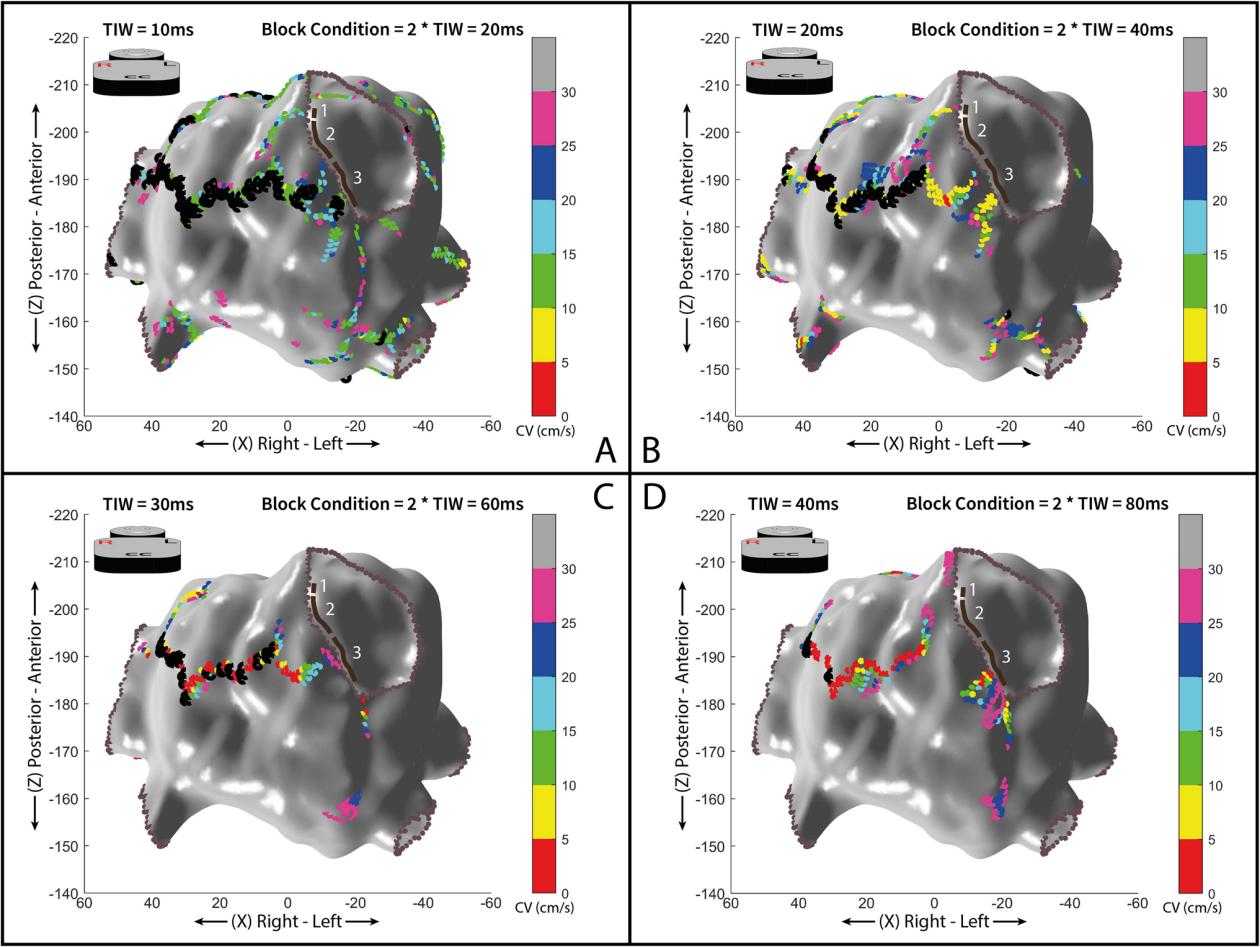
**A** to **D** 3D maps of conduction velocities (CV) of the left atrium during perimitral atrial flutter applying different TIW sizes (A: 10 ms, B: 20 ms, C: 30 ms, and D: 40 ms). Color-coded dots show areas with slow (≤ 30 cm/s) and different CVs. Black dots highlight conduction block lines. Each axis’ values are expressed as millimeters. White numbers, from 1 to 3, indicate areas inside the critical isthmus of the AFL with different CVs. TIW set to 10 and 20 ms allows to better identify the critical isthmus of the AFL with inside alternating areas of low and faster CV. TIW of 30 and 40 ms results too large with the consequent minor ability to spatially identify the small area of the critical isthmus.

On the contrary, the identification of the blocks may be influenced by the TIW size chosen. Blocks were overestimated with TIW at 10 ms and underestimated with TIW at 40 ms. Local blocks are identified when there was at least a “jump” of one isochronal map area, which, in the case of TIW = 20 ms, corresponding to 40 ms. By using a TIW size of 20 ms, it was possible to identify local blocks of clinical interest which were not directly captured by the EAM (see Figs. 6 and 7).

Differences in CV were found applying four different TIW by analyzing the confined areas of the critical isthmuses either for AFL or VT.

In Tables 2 and 3, we reported data on the estimated CV for the identified areas of interest (critical isthmuses of the circuits) in the atypical (perimitral) atrial flutter and VT clinical cases, across the different values of TIW size.

#### AFL

**Table 2:** Atypical (perimitral) AFL CV values (cm/s)

TIW size	1. Entry zone	2. Central zone	3. Exit zone
10 ms	16.90 ± 5.70	80.32 ± 33.29	15.25 ± 4.60
20 ms	11.69 ± 6.42	55.10 ± 20.76	18.03 ± 7.73
30 ms	18.59 ± 8.14	47.08 ± 5.70	13.43 ± 8.85
40 ms	20.04 ± 9.91	37.95 ± 6.12	16.73 ± 8.25
ANOVA	*p* = 0.58	*p* < 1E − 3	*p* < 1E − 3

In this table, mean conduction velocities for the AFL circuit are specified for each isthmus zone (entry, central, and exit zone). Each table line shows the resulting CV for a different choice of TIW. The bottom line shows statistical significance in the differences in CV values due to a different TIW chosen

#### VT

**Table 3:** VT CV values (cm/s)

TIW size	1. Entry zone	2. Central deceleration zone	3. Central acceleration zone	4. Exit zone
10 ms	14.12 ± 5.22	13.74 ± 4.14	41.87 ± 8.12	15.35 ± 4.96
20 ms	8.84 ± 6.03	6.13 ± 1.34	91.29 ± 35.95	9.35 ± 5.49
30 ms	8.42 ± 7.44	8.23 ± 6.32	NaN	12.16 ± 6.80
40 ms	8.24 ± 7.61	3.74 ± 2.14	NaN	7.96 ± 3.86
ANOVA	*p* < 1E − 3	*p* < 1E − 3	*p* < 1E − 3	*p* < 1E − 3

In this table, mean conduction velocities for the VT circuit are specified for each isthmus zone (entry, central zones, and exit zone). Each table line shows the resulting CV for a different choice of TIW. The bottom line shows statistical significance in the differences in CV values due to a different TIW chosen

## Discussion

The main finding of the study is the creation of an automated technique for CV estimation based on the isochronal activation time of the heart chambers.

This technique is applicable both during cardiac arrhythmias and sinus/paced rhythm, helping to discover pathologic areas with slower CV and granting a clinical advantage by integrating the activation mapping with a visual CV map.

### Clinical implications

During atrial or ventricular arrhythmias, the automated method for CV mapping adds clinical information on the areas with slow and heterogeneous wavefront propagation or about functional/anatomical conduction blocks, both crucial to maintaining the arrhythmia’s circuit.

The most common ablation scheme for perimitral atrial flutter is generally a lesion line between the left inferior pulmonary vein and the lateral segment of the mitral annulus without taking into account the location of the abnormal substrate supporting the arrhythmia. The cases we reported represent examples to show how the proposed method may be useful in clinical practice since it could help to choose the patient in which an anterior ablation approach could be better than a classic posterior-lateral line. In our case of perimitral atrial flutter, indeed, the area of slow conduction is well identified and located in the anterior wall.

Moreover, additional ablation inside the coronary sinus or the alcoholization of the Marshall’s vein is often required to obtain a transmural lesion [21]. The ability to detect areas of slow conduction, which are peculiar for each individual patient, offers a tool for individualized lesion set involving the abnormal tissue maintaining the arrhythmia, potentially helping to reduce recurrences and reducing the number of necessary ablations.

In addition, Fig. 2 shows the difference in slow CV areas between a healthy LA and that of a patient with AF history, in sinus rhythm. The atrium of the subject suffering from AF shows multiple areas of slow conduction as a marker of conduction disturbances potentially favoring atrial fibrillation/flutters maintenance. This information could support the decision to extend the ablation beyond the pulmonary vein isolation.

CV map of the endocardial/epicardial ventricles’ surfaces during sinus rhythm could be useful to discover functional/anatomical substrate favoring poorly tolerated ventricular arrhythmias. The proposed method could allow for automatically localizing and quantifying DZs with the consequent clinical advantages already reported by Tung et al. [22]. Furthermore, our method may also facilitate the application of the clinical findings by Anter et al. [23]: the intraoperative study of the wavefront propagation during either SR or apical pacing is a method to confirm the presence of slow CV areas impacting the genesis and maintenance of VT circuits.

The analysis of CV in the case of tolerated and mappable ventricular tachycardia, as reported in Fig. 5 panel B, reveals left ventricle areas of slow conduction at the entrance and exit of the VT isthmus region. The VT isthmus is automatically confined and characterized by inhomogeneous CV.

The common ablation strategy usually consists of an isthmus transection at any site. Our methodology could help to better define and target the isthmus area with the slowest conduction, but it can also reveal the extension of slow CV areas localized at the entrance or exit sites, which could be the substrate for other circuits and multiple VT morphologies.

### Analogies and differences with other methods

Since the heterogeneity of the CV is crucial for arrhythmogenicity, multiple approaches for CV estimation have been proposed by investigators. Weber et al.[6] developed a technique to analyze the activation pattern across the whole set of a catheter’s electrodes based on the model where a plane excitation wavefront travels across a circular multipolar catheter. This method is not based on activation time maps of the whole atrium, but it may be considered a single-shot analysis of an individual wavefront passing across the catheter’s electrodes. The wavefront directions are measured in the local coordinate system of the catheter. If the catheter is rotated, the results change even if the activation pattern remains the same. In addition, this technique is not applicable when there are two colliding wavefronts or for different mapping catheter types since the cosine model is not valid anymore. In our study instead, after the acquisition of all the activation timings and points’ spatial coordinates, CV can be calculated just by defining the TIW size.

Doshi et al. [4] developed a semi-automated approach based on the single vector method to measure the CV between two manually selected points along the perceived longitudinal axis of wave-front propagation and perpendicularly to it. This approach grants measurement of CV along multiple axes, but it requires that the user defines the location of a reference starting point and a terminal point on the activation map and estimates CV between those points. Our approach does not require user intervention, and the CV map of the heart chamber is automatic.

Recently, Roney et al. [8] published a strategy based on a wave-front-free approach. The CV estimation relies on random sampling in selected areas and multiple calculations between node pairs. Propagation wavefront is instead crucial in our approach, and node pairs are not randomly chosen across the map: we link points from two adjacent isochrones, excluding node pairs pertaining to the same wave-front, thus allowing to avoid CV overestimation.

We demonstrated these differences by applying Roney’s method to the presented clinical cases. For instance, in the AFL case, our method returns a CV mean value of around 12.4 cm/s, while Roney’s method is around 18.0 cm/s.

Finally, while Roney’s method provides estimated velocities that are very similar in longitudinal and transverse directions, our method can provide more relevant differences between the two, improving accuracy in pinpointing deceleration and acceleration zones in the studied areas.

### Temporal of interest window (TIW) size

No significant differences in CV calculation at different TIW sizes (10, 20, 30, and 40 ms) were found when the data of the global heart chamber map were computed.

Nonetheless, the choice of the TIW size is an important factor for this technique: using values that are too high may result in violations of the assumption of CV uniformity within each MDP (a condition that can occur if the temporal distance between starting and arrival points is too high), while using values that are too low may result in reduced CV estimation accuracy, as determined by the increased relative effect of possible inaccuracies in timing annotations.

Tables 2 and 3, statistical analysis for the atypical AFL and VT cases, document the relevance of the TIW choice when the method is applied to calculate the CV inside the limited areas of the critical isthmuses of the reentrant circuits. This is due mostly to the fact that the CV values for TIW = 10 ms were significantly different from (i.e., higher than) the other cases (20, 30, and 40 ms). Our explanation for this behavior is that, if the TIW size is too small, the number of points within each isochronal map area becomes insufficient to warrant an accurate definition of the minimum distance pathway, whose delineation may suffer from capturing nodes that are within the same front, thus overestimating the MDP.

For this reason, the TIW = 10 ms should not be recommended. However, the implemented method resulted to be robust for the other TIWs, especially in detecting zones of slow conduction. For the presented study, the choice of TIW = 20 ms was made for clinical reasons, as we deemed that the maps with such size could be more easily interpreted.

Raiman and Tung [15] have already focused their research on this field, performing clinical analyses of the isochronal late activation mapping (ILAM). They identified that regions where 3 or more isochrones within an area of 1 cm radius, during sinus rhythm, represented deceleration zones (DZs) as anchor points for VT [15, 22]. The time of activation window was divided into 8 isochronal zones, each showing 12.5% of the total activation time, with a consequent bias due to the variable nature of the TIW size chosen. Indeed, this could lead to variable results in deceleration zone detection. Patients displaying longer activation windows require higher values of CV deceleration to be considered DZs, compared to those with shorter activation windows.

In our study, since the TIW size was set a priori at 20 ms, we minimized this unwanted variability. For example, the numerical value chosen for TIW allowed us to obtain at least 13 different isochronal map areas for the reported peri-mitral AFL case and 26 for the case of VT.

The subdivision into consecutive isochronal map areas is of critical importance for CV estimation since it has an impact on the spatial localization and visualization of possible CV decelerations. Through the isochronal display of tissue activation, it is possible to identify regions of slow conduction, located where the borders of successive isochrone map areas are closer together.

An alternative approach has been developed by Roney et al. [8] who, simplifying the dimensionality of the anatomical mesh, adopted a multidimensional scaling based on a two-dimensional flattening introduced by Zigelman[24]. This method preserves geodesic distances only in the proximity of the pacing locations, and the estimation accuracy decreases further from this location because of heterogeneities in the fiber field.

However, in the cited methodologies, spatial distortions are introduced, leading to an under- or over-estimation of the resulting CV, since the 2-D projection of the 3-D anatomical representation will introduce not negligible approximations.

Different from the research of Roney [8] and Zigelman [24], we maintained the 3D structure of the anatomy to calculate the geodesic distances, preserving the dimensionality of the 3D recording locations. This was deemed necessary as we analyzed the whole anatomy of the chamber, to avoid spatial distortions, especially in areas of greater convexity, such as pulmonary veins.

Following the principle of minimum traveled distance, using a starting and an arrival point for calculations, our method is also able to both simplify the computational complexity of the CV calculation and to exclude from the calculations the points not pertaining to the same propagation branch.

### Limitations

The RHYTHMIA Mapping System annotates the timing of the bipolar electrograms at the EGM component with the highest voltage. Alternative annotation strategies, such as those based on choosing the onset or the offset of the bipolar electrogram, could generate a change of isochronal extension, and consequently, also a different CV map, especially where inhomogeneous and prolonged EGM duration is observed. Sensitivity to potential isochronal time shifts will be investigated once a specific algorithm capable of using different annotation criteria will be available in the system.

The accuracy of the CV map is influenced by the number of annotated points considered reliable for the activation map. In the presented clinical cases, the EAMs density is around 100–150 points/cm^2^, a value that reassured us of the robustness of the method. Finally, if the TIW is shortened to reach higher sensitivity in the detection of areas with slower CV, a denser map is required to have a sufficient number of vertices over the smaller area to reduce errors in CV estimation. The accuracy in CV calculation is then a matter of compromise between TIW size and EAM point density.

The patient population is limited, but the purpose of this paper is to describe the methodology for CV estimation based on isochronal activation, and the clinical cases presented represent only examples of potential clinical applications that may be the object of future studies.

## Conclusions

The proposed software for automated CV estimation allows finding areas with low CV, potentially helping electrophysiologists to plan the ablation strategies. Larger studies are warranted to further confirm our findings.
